# Plastic Evolution Characterization for 304 Stainless Steel by CQN_Chen Model under the Proportional Loading

**DOI:** 10.3390/ma16216828

**Published:** 2023-10-24

**Authors:** Xiang Gao, Songchen Wang, Zhongming Xu, Jia Zhou, Xinming Wan, Hasib Md Abu Rayhan, Yanshan Lou

**Affiliations:** 1 College of Mechanical and Vehicle Engineering, Chongqing University, Chongqing 400044, China; torin_gao@yeah.net (X.G.);; 2 China Automotive Engineering Research Institute Co., Ltd., Chongqing 401122, China; 3 School of Mechanical Engineering, Xi’an Jiaotong University, 28 Xianning West Rd, Xi’an 710049, China; wangsc@stu.xjtu.edu.cn (S.W.);

**Keywords:** anisotropic hardening, plastic evolution, convex

## Abstract

In this paper, the CQN_Chen function is used to characterize the plastic anisotropic evolution of 304 stainless steel (SS304). The uniaxial tensile tests along different loading directions are conducted to experimentally investigate the anisotropic hardening behavior for SS304. The experimental data indicates that the anisotropy of SS304 is weak. The convexity analysis is carried out by the geometry-inspired numerical convex analysis method for the CQN_Chen yield locus during plastic deformation. The Hill48, SY2009 and CQN functions are used as the comparison to evaluate the accuracy of the CQN_Chen function in characterizing plastic evolution. The predicted values are compared with the experimental data. The comparison demonstrates that the CQN_Chen function can accurately characterize anisotropic hardening behavior under uniaxial tension along distinct loading directions and equibiaxial tension. Simultaneously, the CQN_Chen model has the capacity to adjust the yield surface shape between uniaxial tension and equibiaxial tension. The CQN_Chen model is recommended to characterize plastic evolving behavior under uniaxial tension along different directions and equibiaxial tension.

## 1. Introduction

Due to carbon neutrality, lightweight materials are widely used to reduce weight, such as aluminum alloy, magnesium alloy and stainless steel [1]. During the rolling forming process, the textures of these sheet metals form preferred orientation, which leads to anisotropy. It is vital to accurately characterize the plastic anisotropic evolution in sheet-metal forming numerical simulation.

The Hill48 function was the pioneer of anisotropic yield functions [2]. However, the Hill48 function cannot distinguish the difference of yield surface of metals with different crystal structures because of its quadratic form. Hosford [3,4] combined the exponent with the yield surface shapes for body-centered cubic (BCC) and face-centered cubic (FCC) metals. Barlat et al. [5] extended the isotropic function to anisotropy by introducing anisotropic coefficients through a linear transformed tensor. More anisotropic coefficients were introduced through two linear transformed tensors to characterize strongly anisotropic metals and consider r-values, such as Yld2000-2d [6] and Yld2004-18p [7]. Cazacu et al. [8] introduced an orthotropic yield criterion in the form of the principal values of the stress deviator to capture the anisotropy and the asymmetry between tension and compression. He et al. [9] proposed an enhanced constitutive model based on the Yld2000-2d model. Recently, many yield functions were developed based on stress invariants. Cazacu and Barlat [10] extended the isotropic Drucker [11] function to orthotropy. Cazacu and Barlat [12] modelled the asymmetry and anisotropy for pressure insensitive metals. Gao et al. [13] developed a plasticity model with the form of the hydrostatic stress as well as the second and third invariants of the stress deviator. Aretz and Barlat [14] developed a very flexible anisotropic yield function with 27 anisotropic parameters. Yoshida et al. [15] developed an anisotropic yield function in a form of the stress invariants. Hu et al. [16] proposed a normalized stress invariant-based yield criterion to describe both asymmetry and Lankford coefficients. Lou et al. [17] extended the Drucker yield function to anisotropy by linear transformed tensor, and calibrated the yield surface shape control parameters for BCC and FCC materials. When a linear transformed tensor is used to introduce anisotropic coefficients, it is generally necessary to use an optimization algorithm to optimize error functions to calibrate anisotropic coefficients under specific plastic work. Xu et al. [18] reduced the Yld2011-27p function to decrease the number of experiments for the calibration of anisotropic parameters. Therefore, the anisotropic yield functions commonly cannot accurately describe the change of the yield surface with increasing plastic strain under the isotropic hardening.

The anisotropic hardening model captures the evolution of the yield surface by analytically describing the anisotropic coefficient. Namely, the hardening curves under different stress states are directly used to calculate the anisotropic coefficients with the increase of the plastic deformation. The anisotropic hardening was experimentally observed by Choi et al. [19], Khan et al. [20,21,22], Lou et al. [23], etc. Stoughton and Yoon [24] replaced the anisotropic coefficients of the Hill48 function with hardening curves under uniaxial tension along the rolling direction (RD), diagonal direction (DD) and transverse direction (TD) as well as equibiaxial tension, which achieved the accurate description of anisotropic hardening behavior (SY2009). The SY2009 function inherited the characteristic of the Hill48 function. Namely, the shape of the yield surface cannot be adjusted by the SY2009 model. Lee et al. [25] multiplicatively coupled SY2009 with the Hosford72 [26] yield criteria, in which the plastic evolution was characterized by SY2009 function, and the shape of the yield surface was adjusted by the Hosford72 function (CQN). Chen et al. [27] coupled SY2009 with Drucker function to characterize anisotropic hardening for BCC and FCC metals (CQN_Chen). Hou et al. [28] coupled a quadratic asymmetric yield function with a non-quadratic function by multiplication to characterize the anisotropic–asymmetric hardening behavior and adjust the yield surface shape. Hou et al. [29] proposed an anisotropic hardening model by coupling an asymmetric Hill48 function with an isotropic stress-invariant-based yield function. Hu et al. [30] coupled a fourth order polynomial yield criterion with a non-quadratic yield function under associated flow rule to describe the evolution of anisotropic yielding behavior analytically. Zhang and Lou [31] characterized the evolving plastic behavior by coupling an enhanced pDrucker function and SY2009 model. Zhou et al. [32] proposed a new analytical asymmetric yield criterion base on the SY2009. In addition to constructing anisotropic hardening model through multiplicative coupling method, a direct analytical description of the yield function is developed by the hardening curves or r-value evolution of different stress states to describe the anisotropic hardening. Hu et al. [33] analytically described the Yoon2014 [34] function by considering anisotropic hardening. Hu et al. [35] analytically described polynomial yield criterion by considering both plane strain and pure shear states. Lou et al. [36] converted the stress-invariant-based function into stress triaxiality, Lode parameter and von Mises stress forms, and analytically described the parameters through four of the five stress states. Hou et al. [37] proposed a constitutive model to accurately describe the anisotropic behaviors of sheet metals in terms of the yield stress and plastic flow under plane strain loading. Lou and Yoon [38] constructed an anisotropic-asymmetric hardening model by the additive coupling of two Hill48 functions with Lode-dependent weight functions. The more parameters in the analytical description of the yield function, the more anisotropic hardening behaviors of the stress states can be characterized.

The anisotropic models are widely used to predict the formation of sheet metals. Clausmeyer and Svendsen [39] compared two of the models for anisotropic hardening and yield surface evolution in BCC sheet steels. Min et al. [40] used the Yld89 [41] function to consider anisotropic hardening and sheet anisotropy for a multi-phase 980 MPa steel. Li et al. [42] characterized the anisotropic hardening behavior of metals using the interpolation method. Hao and Dong [43] proposed an interpolation-based anisotropic yield and hardening models. Fu et al. [44] proposed a method to simultaneously identify the anisotropic yield and hardening parameters from a single test. Li et al. [45] developed an improved yield criterion to characterize the anisotropic and tension-compression asymmetric behavior of magnesium alloy. Lou et al. [46] developed a reduced form to the Yld2004-18p function to decrease the experimental cost for the calibration of anisotropic parameters. Wang et al. [47] investigated the earing characteristics of the 6K21 aluminum alloy in a circular-cup deep drawing by virtue of experiments and multi-scale simulations. Hou et al. [48] described the evolving yield surfaces of dual-phase steels. Mu et al. [49] characterized the anisotropic hardening and evolution of r-values for sheet metal based on the evolving non-associated Hill48 model. Du et al. [50] characterized the asymmetric evolving yield and flow of the 6016-T4 aluminum alloy and DP490 steel by the several existing asymmetric yield criteria under the associated and non-associated flow rules. Mamros et al. [51] captured the plastic anisotropy evolution of stainless steel 316L under proportional loadings. Yang et al. [52] investigated the anisotropic plastic flow of low/medium carbon steel plates in different loading conditions. Lee et al. [53] utilized the evolving Hill48 function to describe the evolution of the distortional yield surface of the Al6014-T4 alloy. Gawad et al. [54] developed an evolving plane stress yield criterion based on crystal plasticity virtual experiments.

This research investigates the plastic anisotropic evolution of 304 stainless steel (SS304) by using the CQN_Chen function. The uniaxial tensile tests along RD, DD and TD are conducted. The convex domain of CQN_Chen function is determined by the geometry-inspired numerical convex analysis (GINCA) method with the increasing plastic evolution. The Hill48, SY2009 and CQN models are selected as a comparison to reveal the capability of CQN_Chen model in characterizing anisotropic hardening and adjusting yield surface shape. The experimental yield stresses are fully compared to the predicted values by the four models with the increase of the plastic deformation, including 3D plane stress yield surfaces $\left(\sigma_{xx},\sigma_{yy},\sigma_{xy}\right)$, 2D yield locus under biaxial loading, predicted uniaxial tensile yield stresses, and hardening curves under uniaxial tension along RD, DD and TD as well as equibiaxial tension, which is used to evaluate the accuracy of CQN_Chen function in describing plastic evolution.

## 2. CQN_Chen Anisotropic Hardening Model

### 2.1. CQN_Chen Anisotropic Hardening Model

The CQN_Chen function is proposed by Chen et al. [27], as shown in Equation (1). The CQN_Chen is formulated by coupling SY2009 and Drucker function. Hence, the function can accurately capture anisotropic hardening and adjust the yield surface shape for BCC and FCC metals under the proportional loading conditions. Meanwhile, the function is convenient for numerical simulation and has high simulation efficiency.

(1) $$f_{CQN\_Chen}\left(\sigma ,\bar{\lambda }\right)={\left[f_{Hill48}\left(\sigma ,\bar{\lambda }\right)•f_{Druc\ker}\left(\sigma \right)\right]}^{\frac{1}{8}}$$

with

(2) $$\begin{array}{c} f_{Hill48}\left(\sigma ,\bar{\lambda }\right)=F\left(\bar{\lambda }\right){\left(\sigma_{22}-\sigma_{33}\right)}^{2}+G\left(\bar{\lambda }\right){\left(\sigma_{33}-\sigma_{11}\right)}^{2}+H\left(\bar{\lambda }\right){\left(\sigma_{11}-\sigma_{22}\right)}^{2}\\ +2L\left(\bar{\lambda }\right)\sigma_{23}^{2}+2M\left(\bar{\lambda }\right)\sigma_{31}^{2}+2N\left(\bar{\lambda }\right)\sigma_{12}^{2} \end{array}$$

(3) $$f_{Druc\ker}\left(\sigma \right)=a\left(J_{2}^{3}-cJ_{3}^{2}\right)$$

where $\bar{\lambda }$ denotes the plastic compliance factor; $\left(\sigma_{11},\sigma_{22},\sigma_{33},\sigma_{12},\sigma_{23},\sigma_{13}\right)$ present Cauchy stress components; $J_{2}$ and $J_{3}$ indicate the second and third invariants of stress deviator, respectively; c controls the yield surface shape, and c=1.5776 for BCC and c=2.5116 for FCC; $a=\frac{729}{27-4c}$; and $F\left(\bar{\lambda }\right)$, $G\left(\bar{\lambda }\right)$, $H\left(\bar{\lambda }\right)$, $L\left(\bar{\lambda }\right)$, $M\left(\bar{\lambda }\right)$ and $N\left(\bar{\lambda }\right)$ are anisotropic parameters of the Hill48 function. The six parameters are functions of the hardening curves along different loading directions, which is depicted as follows:

(4) $$F\left(\bar{\lambda }\right)=\frac{1}{2}\left(\frac{1}{{\left[\sigma_{90}\left(\bar{\lambda }\right)\right]}^{8}}+\frac{1}{{\left[\sigma_{b}\left(\bar{\lambda }\right)\right]}^{8}}-\frac{1}{{\left[\sigma_{0}\left(\bar{\lambda }\right)\right]}^{8}}\right)$$

(5) $$G\left(\bar{\lambda }\right)=\frac{1}{2}\left(\frac{1}{{\left[\sigma_{0}\left(\bar{\lambda }\right)\right]}^{8}}+\frac{1}{{\left[\sigma_{b}\left(\bar{\lambda }\right)\right]}^{8}}-\frac{1}{{\left[\sigma_{90}\left(\bar{\lambda }\right)\right]}^{8}}\right)$$

(6) $$H\left(\bar{\lambda }\right)=\frac{1}{2}\left(\frac{1}{{\left[\sigma_{0}\left(\bar{\lambda }\right)\right]}^{8}}+\frac{1}{{\left[\sigma_{90}\left(\bar{\lambda }\right)\right]}^{8}}-\frac{1}{{\left[\sigma_{b}\left(\bar{\lambda }\right)\right]}^{8}}\right)$$

(7) $$L\left(\bar{\lambda }\right)=M\left(\bar{\lambda }\right)=N\left(\bar{\lambda }\right)=\frac{1}{2}\left(\frac{4}{{\left[\sigma_{45}\left(\bar{\lambda }\right)\right]}^{8}}-\frac{1}{{\left[\sigma_{b}\left(\bar{\lambda }\right)\right]}^{8}}\right)$$

where $\sigma_{0}$, $\sigma_{45}$, $\sigma_{90}$ and $\sigma_{b}$ are the yield stresses under uniaxial tension along the RD, DD and TD as well as equibiaxial tension, respectively.

### 2.2. Convexity Analysis

**Figure 1:**
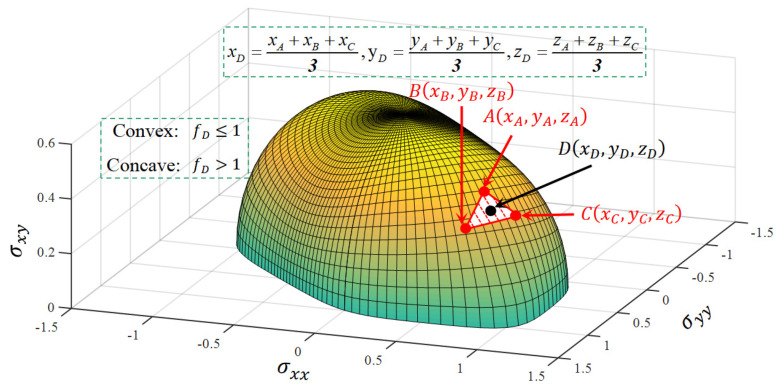
Schematic diagram of convexity analysis of yield surface under plane stress space by GINCA.

Equations (2) and (3) observe that the c and $\bar{\lambda }$ values are related to the convexity of the CQN_Chen yield surface. Namely, the c adjusts the yield surface curvature, and the flow stresses under distinct plastic strain are obtained by the $\bar{\lambda }$ to compute the anisotropic parameters of Hill48 function. The convexity of the yield locus is usually validated by the Hessian matrix with the positive semi-definite. The Hessian matrix is obtained by calculating the second order partial derivative of the yield function, and the convexity of the yield surface is judged according to the eigenvalue of the Hessian matrix. It is inconvenient and time-consuming to verify the convexity of the yield locus during the plastic deformation process. Lou et al. [36] proposed a geometry-inspired numerical convex analysis (GINCA) method to determine the convexity of the yield surface by calculating the equivalent stress, as depicted in Figure 1. The points A, B and C are on the 3D plane stress yield surface. Point D is located at the center of the infinitesimal curved surface $\tilde{ABC}$. The coordinates of points A, B and C are presented as $\left(x_{A},y_{A},z_{A}\right)$, $\left(x_{B},y_{B},z_{B}\right)$ and $\left(x_{C},y_{C},z_{C}\right)$, respectively. Therefore, the coordinate of point D is denoted as $\left(\left(x_{A}+x_{B}+x_{C}\right)/3,\left(y_{A}+y_{B}+y_{C}\right)/3,\left(z_{A}+z_{B}+z_{C}\right)/3\right)$. Due to points A, B and C being located on the yield surface, the equivalent stresses at these points are $f_{A}=f_{B}=f_{C}=1$. The equivalent stress at point D needs to be calculated to judge the convexity of the $\tilde{ABC}$. If point D is located inside the curved surface $\tilde{ABC}$, then $\tilde{ABC}$ is convex. Namely, the curved surface $\tilde{ABC}$ is convex if $f_{D}\leq 1$. On the contrary, the curved surface $\tilde{ABC}$ is concave if $f_{D}>1$. It is convenient to use the GINCA method to analyze the convexity of the yield surface during the plastic evolving process. It is only necessary to calculate the equivalent stress of the yield function without the computation of the second–order partial derivative compared to the complicated Hessian matrix.

## 3. Plastic Evolution Characterization for SS304

### 3.1. Uniaxial Tensile Experiment

**Figure 2:**
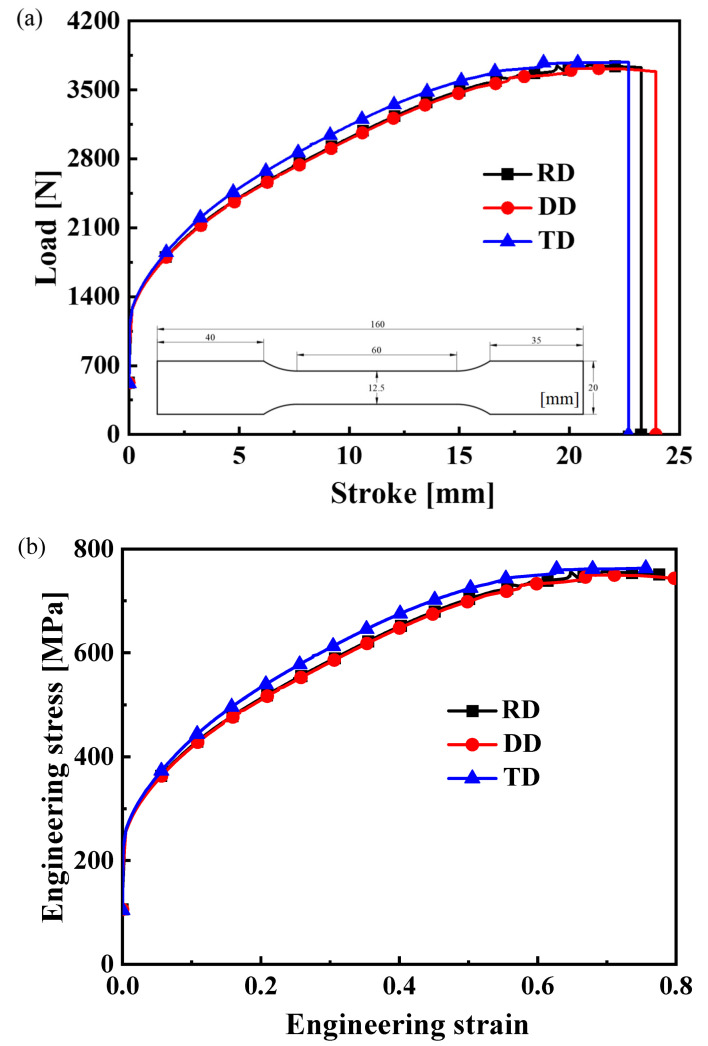
Mechanical curves of uniaxial tension along the RD, DD and TD for SS304: (**a**) load–stroke curve; (**b**) engineering stress–engineering strain curve.

The CQN_Chen function is analytically described by the hardening curves of uniaxial tension along the RD, DD and TD as well as equibiaxial tension for SS304 in Equations (4)–(7). Hence, the dog–bone specimens with a thickness of 0.4 mm are cut by a laser cutting machine along the RD, DD and TD for uniaxial tension. The dimensions of the dog–bone specimen are shown in Figure 2. The uniaxial tensile tests are under quasi–static loading with a tensile speed of 3.6 mm/min. The uniaxial tensile test is repeated five times in each direction to ensure the reliability of the experimental results. The surface of the specimen is sprayed with speckle uniformly, and the deformation process is recorded by the XTOP digital image correction system. The virtual extensometer is set to 30 mm to obtain the load–stroke curve in the longitudinal direction, as presented in Figure 2a. 

**Figure 3:**
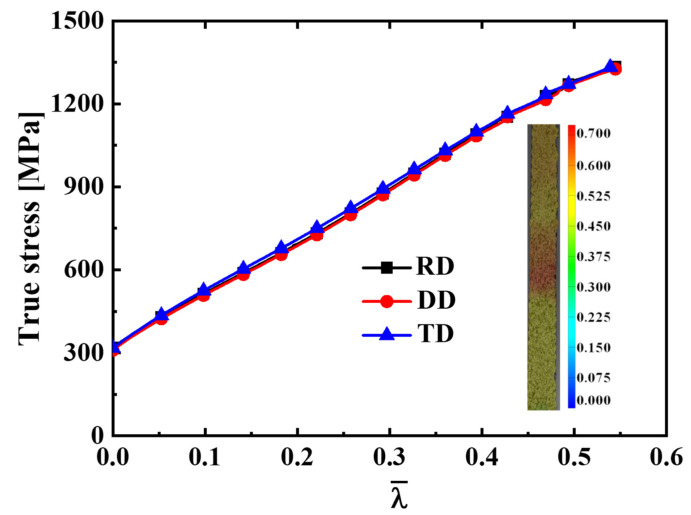
The hardening curves of unaixial tension along theRD, DD and TD for SS304.

Figure 2a shows that the load–stroke curves in the RD and DD are almost identical, and the load–stroke curve in the TD is slightly higher than that in the RD and DD. The engineering stress–engineering strain curve is depicted in Figure 2b. According to the principle of plastic work equivalence, the uniaxial tensile hardening curves along different directions are converted into functions of $\bar{\lambda }$ in order to calculate the parameters of the CQN_Chen function, as shown in Figure 3. The hardening curve of TD is slightly higher than that of RD and DD at $0.15\leq \bar{\lambda }\leq 0.35$. The anisotropy is not obvious for SS304. The equivalent strain field in Figure 3 shows that the equivalent plastic strain of SS304 at fracture is about 0.6, which indicates that SS304 has good plastic deformation capacity. The uniaxial tensile hardening curves along the RD, DD and TD are fitted by the Hockett–Sherby/Hollomon hardening model. The parameters of the hardening law are listed in Table 1. The yield stress under equibiaxial tension is assumed as $\sigma_{b}=\left(\sigma_{0}+2\sigma_{45}+\sigma_{90}\right)/4$.

### 3.2. Anisotropic Evolution Characterization of SS304

**Figure 4:**
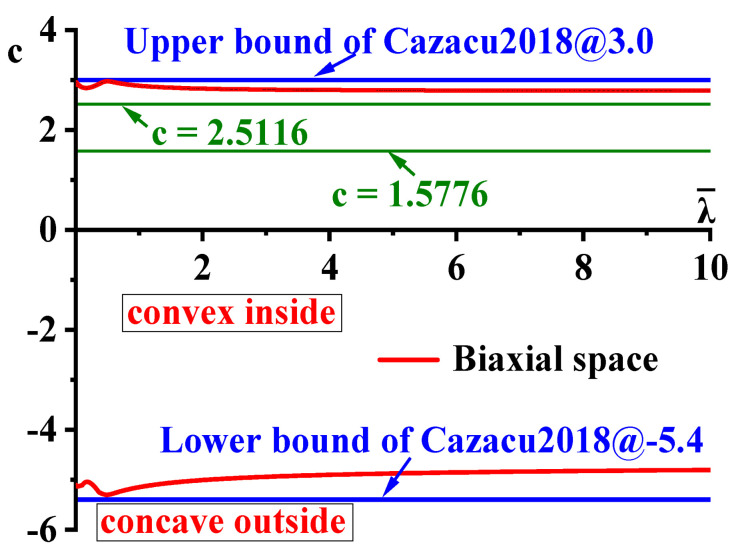
The limit values of the parameter c of CQN_Chen function computed by GINCA method under biaxial loading space for SS304.

The convex domain of the CQN_Chen yield surface is investigated by using the GINCA method under 3D–plane stress space with plastic evolution. Under the assumption of isotropy, the CQN_Chen function is reduced to a Cazacu2018 function [55]. Hence, the convex domain of the CQN_Chen function should be within the convex domain of the Cazacu2018 function. The comparison between the convex domain of CQN_Chen and the convex domain −5.4 ≤c≤3 of the Cazacu2018 function is shown in Figure 4. The blue solid line and the red solid line in Figure 4 represent the convex domains of the Cazacu2018 and CQN_Chen functions, respectively. The comparison indicates that the upper and lower limits of parameter c for CQN_Chen are located in the convex domain of Cazacu2018 function. The parameter c of the CQN_Chen function is recommended to be 1.5776 for SS304 of BCC metal. c=1.5776 is always in the convex domain of the parameter c at $0\leq \bar{\lambda }\leq 10$. Therefore, the CQN_Chen yield surface remains convex during plastic deformation for SS304.

**Figure 5:**
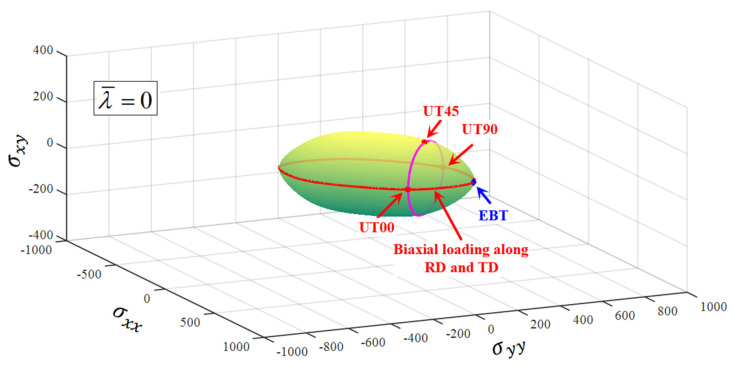

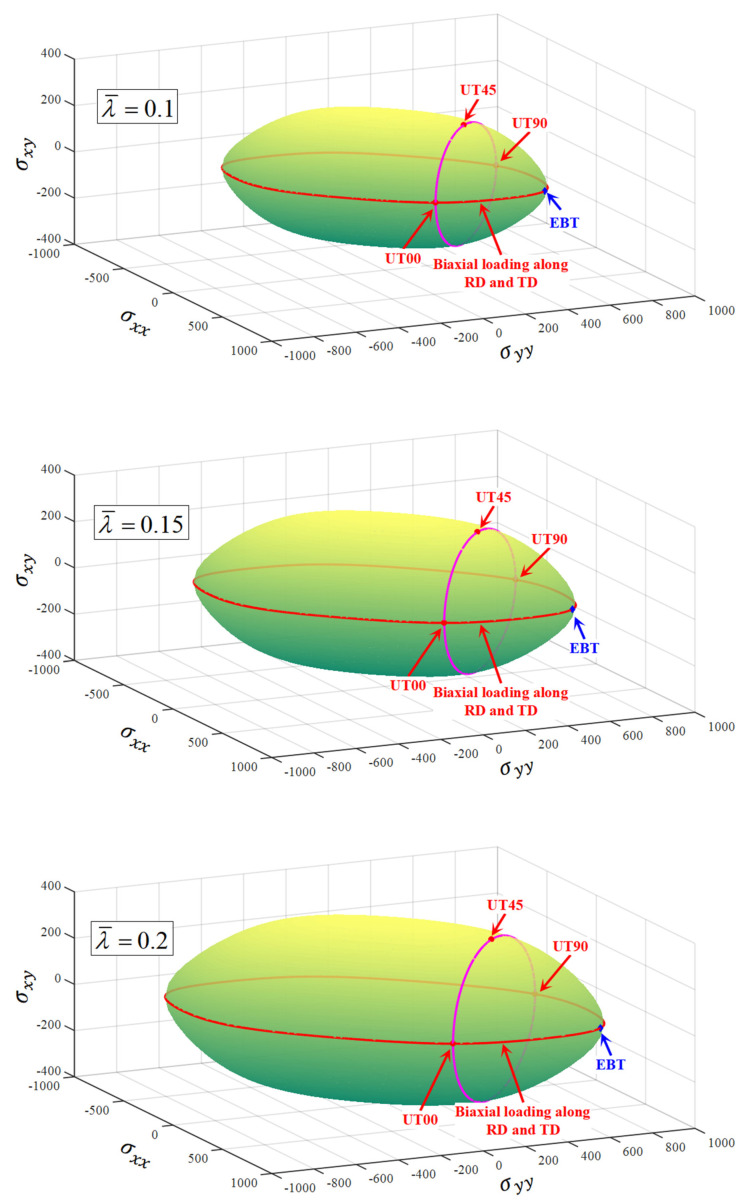
The evolution of 3D plane stress yield surface of CQN_Chen function at different $\bar{\lambda }$ for SS304.

Figure 5 presents the 3D plane stress yield surfaces predicted by the CQN_Chen function at $\bar{\lambda }=0$, 0.1, 0.15 and 0.2. The yield locus evolves with an increasing $\bar{\lambda }$ value. The experimental yield stresses are precisely located on the yield surfaces for uniaxial tension along the RD, DD and TD as well as equibiaxial tension at different $\bar{\lambda }$. This is because these yield stresses are used to analytically describe the parameters of the CQN_Chen function. The anisotropic hardening behavior for SS304 is accurately characterized by the CQN_Chen model.

**Figure 6:**
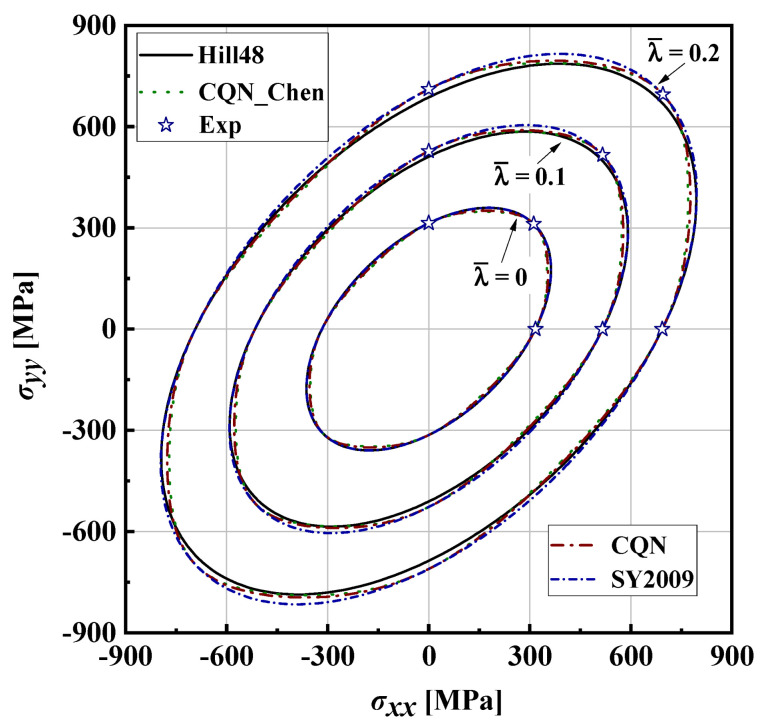
Comparison of 2D yield surfaces predicted by different models with experimental data for SS304.

**Figure 7:**
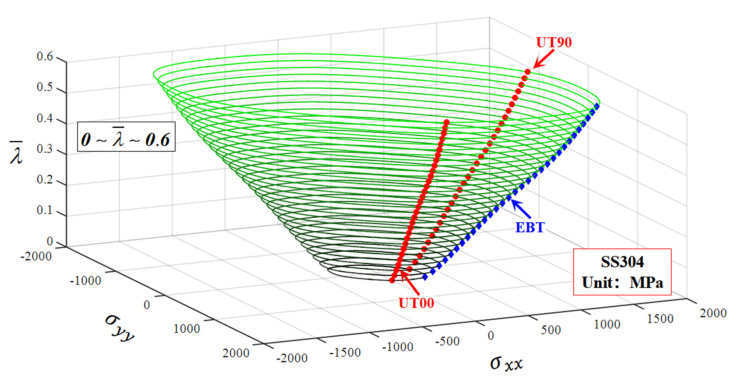
The 2D yield surface evolution predicted by the CQN_Chen function at $0\leq \bar{\lambda }\leq 0.6$ with an interval of 0.02 for SS304.

In order to describe the comparison more intuitively between the predicted yield stresses and the tested values, the 2D yield surface under biaxial loading space is extracted, which is denoted by the solid red line highlighted by <Biaxial loading along RD and TD> in Figure 5. The Hill48 anisotropic function, SY2009 and CQN anisotropic hardening models are selected as a comparison to evaluate the ability of the CQN_Chen model to characterize anisotropic hardening and adjust yield surface curvature. Figure 6 shows the comparison of 2D yield surfaces predicted by different models with experimental data under biaxial loading space. For the Hill48 function with the assumption of isotropic hardening, only the 2D yield surface at $\bar{\lambda }=0$ accurately captures the experimental yield stresses of equibiaxial tension and uniaxial tension along the RD, DD and TD. The uniaxial tensile yield stress along the RD is accurately predicted as the plastic strain increases. However, the predicted yield stresses are underestimated compared to the experimental values under unaixial tension along the TD, and to equibiaxial tension with the increase in plastic deformation. For the three anisotropic hardening models, the predicted yield surfaces at different plastic strain all precisely pass the experimental yield stresses for uniaxial tension along RD and TD as well as equibiaxial tension. The predicted yield surfaces of the CQN and CQN_Chen functions are almost identical. Compared to the predicted yield surface of CQN and CQN_Chen, the predicted yield stresses of SY2009 are significantly smaller than that of CQN and CQN_Chen under plane strain tension along the RD and TD as well as shear along DD. This is because the SY2009 function with the quadratic form cannot distinguish the yield surface differences of metals with different crystal structures. Figure 7 presents 2D yield surface evolution predicted by the CQN_Chen function at $0\leq \bar{\lambda }\leq 0.6$ with an interval of 0.02. The predicted yield surfaces by CQN_Chen are in good agreement with the experimental data of uniaxial tension along the RD and TD as well as the equibiaxial tension at different $\bar{\lambda }$ values. The CQN_Chen function accurately modeled anisotropic hardening behavior and adjusted the shape of the yield surface for SS304.

**Figure 8:**
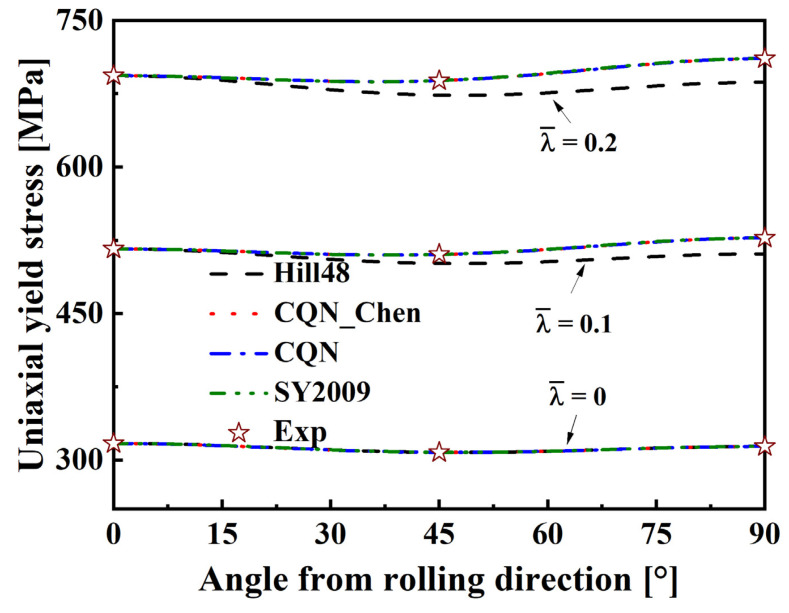
Comparison of uniaxial tensile yield stresses along distinct loading directions predicted by four models with experimental values.

The solid pink lines in Figure 5 are extracted and represents the predicted uniaxial tensile yield stresses along the different directions. Figure 8 indicates the comparison of experimental data with the uniaxial tensile yield stresses along distinct loading directions predicted by the Hill48, SY2009, CQN and CQN_Chen models. The uniaxial tensile yield stresses predicted by the four models accurately pass the experimental values of uniaxial tension along the RD, DD and TD at $\bar{\lambda }=0$. With the increase in plastic strain, the uniaxial tensile yield stresses along distinct loading directions predicted by the Hill48 function are significantly lower than the tested values. However, the uniaxial tensile yield stress along the RD can be accurately predicted by the Hill48 function. The predicted uniaxial tensile yield stresses along different directions nearly overlap for the three anisotropic hardening models. These three models accurately capture the experimental uniaxial tensile yield stresses along RD, DD and TD. The uniaxial tensile yield stresses along RD, DD and TD are precisely characterized at the different plastic strain by CQN_Chen for SS304.

**Figure 9:**
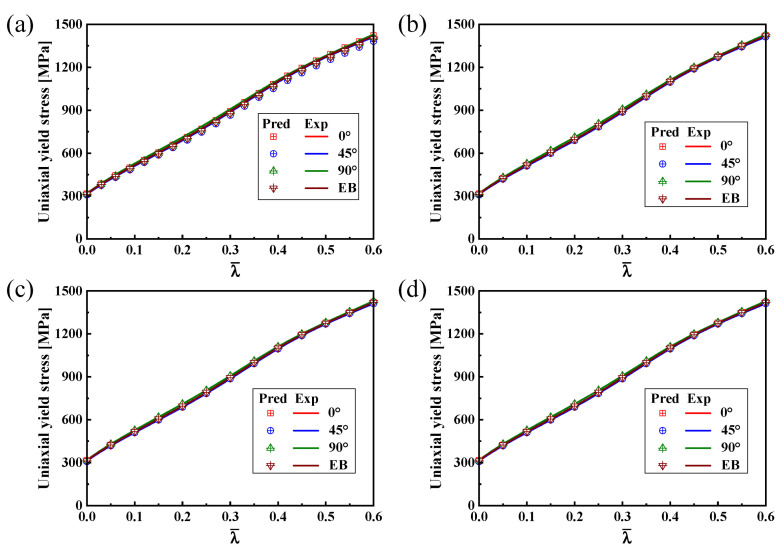
Comparison of hardening curves predicted by different models with experimental data under equibiaxial tension and uniaxial tension along the RD, DD and TD for SS304: (**a**) Hill48 function; (**b**) SY2009; (**c**) CQN; (**d**) CQN_Chen.

**Figure 10:**
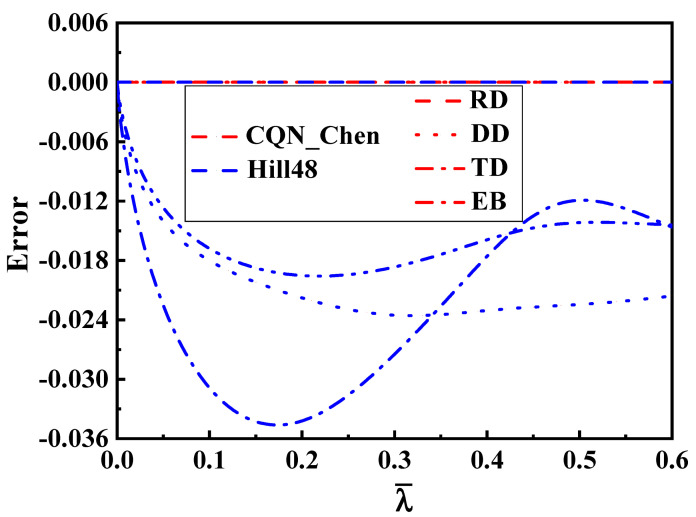
Errors of hardening curves predicted by different functions under equibiaxial tension and uniaxial tension along the RD, DD and TD for SS304.

Figure 9 illustrates the comparison of predicted and tested values for the hardening curves under uniaxial tension along the RD, DD and TD as well as equibiaxial tension. The comparison demonstrates that the four hardening curves are accurately predicted by the SY2009, CQN and CQN_Chen functions. The Hill48 function can only accurately characterize the uniaxial tensile hardening curve along the RD under isotropic hardening. Equation (8) is used to quantitatively evaluate the prediction accuracy of different models for the four hardening curves, as depicted in Figure 10. Consistent with the above analysis, the predicted errors of four hardening curves are zero for the SY2009, CQN and CQN_Chen models. The maximum predicted error of the Hill48 function is about 0.035 for uniaxial tension along TD. Meanwhile, the predicted errors of the Hill48 function are less than zero for the hardening curves of uniaxial tension along the DD and TD as well as equibiaxial tension, which indicates that the predicted yield stresses by the Hill48 function is underestimated under these stress states. The CQN_Chen function accurately describes the hardening behavior for equibiaxial tension and uniaxial tension along the RD, DD and TD.

(8) $$Error=\frac{\left(\sigma_{pred}-\sigma_{\exp}\right)}{\sigma_{\exp}}$$

where $\sigma_{pred}$ and $\sigma_{\exp}$ present the yield stresses of the experiment and prediction, respectively.

## 4. Conclusions

The uniaxial tensile tests along distinct loading directions are conducted to investigate the anisotropic hardening behaviors for SS304. The CQN_Chen function is used to characterize the plastic evolution under uniaxial tension along the RD, DD and TD as well as equibiaxial tension for SS304. The convex domain is verified by GINCA method for the CQN_Chen yield surface. The Hill48, SY2009 and CQN models are selected as the comparison to evaluate the accuracy of CQN_Chen function in describing anisotropic hardening. The predicted values of the four models are compared with the experimental data, including the 3D plane stress yield surface, 2D yield locus under biaxial loading space, the predicted uniaxial tensile yield stresses along different loading directions and the predicted hardening curves. The results shows that the anisotropy of SS304 is not obvious. The CQN_Chen function can accurately model the anisotropic evolution under uniaxial tension along the RD, DD and TD as well as equibiaxial tension with increasing plastic strain. Meanwhile, the yield surface difference are distinguished for BCC and FCC metals. The CQN_Chen model is recommended to characterize plastic evolving behavior for BCC and FCC metals under proportional loading condition.

## Figures and Tables

**Table 1:** The parameters of the Hockett–Sherby/Hollomon hardening law for SS304 uniaxial tensile hardening curves.

Uniaxial Tension	$\sigma \left(\bar{\lambda }\right)=A-B\exp\left(-C{\bar{\lambda }}^{b}\right)+K{\bar{\lambda }}^{n}$						Fitted Error
	A (MPa)	B (MPa)	C	b	K (MPa)	n	
RD	465.291	148.280	67.908	4.172	1499.145	0.878	8.1 × 10^−4^
DD	459.826	151.990	61.747	4.068	1481.643	0.866	9.1 × 10^−4^
TD	417.364	103.482	107.677	4.491	1577.881	0.870	5.1 × 10^−4^

## Data Availability

The data presented in this study are available upon request from the corresponding author.
